# Risk-Guided Personalized Care to Prevent Bronchopulmonary Dysplasia: A Real-World Implementation Study

**DOI:** 10.3390/jpm16060303

**Published:** 2026-06-03

**Authors:** Avram R. Shack, Tapas Kulkarni, Alyssa Hawley, Jessy Jagpal, Maninder Janda, Stephanie Glegg, Uthaya Kumaran Kanagaraj, Michael Castaldo, Julia K. Charlton, Jessie van Dyk, Emily Kieran, Souvik Mitra, Horacio Osiovich, Deepak Manhas, Kanekal S. Gautham, Sandesh Shivananda

**Affiliations:** 1 Division of Neonatology, Department of Pediatrics, University of British Columbia, Vancouver, BC V6T 1Z4, Canada; avram.shack@cw.bc.ca (A.R.S.); tapas.kulkarni@cw.bc.ca (T.K.);; 2 BC Women’s Hospital + Health Centre and BC Children’s Hospital, Vancouver, BC V6H 3V4, Canada; 3 Neonatal Program, BC Women’s Hospital + Health Centre, Vancouver, BC V6H 3V4, Canada; 4 Department of Occupational Science and Occupational Therapy, University of British Columbia, Vancouver, BC V6T 2B5, Canada; 5 Department of Pediatrics, McGovern Medical School, University of Texas Health Sciences Center at Houston, Houston, TX 77030, USA

**Keywords:** neonates, bronchopulmonary dysplasia, implementation strategies, personalized medicine, RE-AIM framework, risk stratification

## Abstract

**Background/Objectives:** Bronchopulmonary dysplasia (BPD) remains a major morbidity among extremely premature infants, with variability in the application of evidence-based interventions between and within neonatal intensive care units (NICUs). We evaluated a multi-component, risk-guided personalized implementation strategy for BPD prevention in a real-world setting. **Methods:** We conducted a prospective observational study of infants <29 weeks’ gestation at birth admitted to a quaternary NICU. The intervention combined risk stratification and structured longitudinal care planning rounds (LCPRs) that included standardized documentation, multidisciplinary facilitation, and associated continuous quality improvement strategies. Implementation outcomes were assessed using the reach, effectiveness, adoption, implementation, and maintenance (RE-AIM) framework. Secondary outcomes included care processes, provider-reported measures, and exploratory clinical outcomes. **Results:** Over six months, 41 infants were included. Risk stratification was consistently applied and all fifteen high-risk infants received LCPR, demonstrating targeted reach. Multidisciplinary participation was broad, with implementation fidelity reflected by consistent screening, structured documentation, and timely care plan execution. Practice standardization was observed, including consistent corticosteroid use (100%), earlier initiation of systemic postnatal steroids (median 16 days), and selective adjunctive therapy use. Providers reported improved teamwork, care coordination, and confidence. Rates of BPD or mortality were comparable between higher-risk infants receiving LCPR and lower-risk infants, despite greater illness severity in the LCPR group. Respiratory severity scores showed a downward trend after implementation, though this did not reach statistical significance (*p* = 0.07). Strategy use continued beyond the study period indicating early sustainability. **Conclusions:** A multi-component, risk-guided implementation strategy can be effectively integrated into routine NICU practice, improving care processes while maintaining clinical outcomes in high-risk infants compared with lower-risk infants.

## 1. Introduction

### 1.1. The Burden of BPD—A Major Morbidity and Public Health Concern

Extreme preterm infants (EPTs), defined as neonates born before 29 weeks’ gestation [1,2,3], represent one of the most vulnerable and resource-intensive populations in neonatal care [4]. Despite advances in management, 12% die and 56% develop bronchopulmonary dysplasia (BPD), defined by the need for respiratory support at 36 weeks’ postmenstrual age (PMA) [5,6]. BPD is associated with prolonged hospitalization in the neonatal intensive care unit (NICU), neurodevelopmental impairment, and long-term healthcare needs, contributing to widening health inequities across the life course [3,7,8]. Additional healthcare costs associated with BPD are estimated to be CA$77,000 per infant (95% CI 75,000–80,000), with lifetime costs surpassing US$700,000 per child [9,10,11,12].

### 1.2. Local Problem, Magnitude, and Context

The BC Women’s Hospital + Health Centre (BCWH) NICU in Vancouver, Canada, admits approximately 70 EPTs annually; these account for nearly half of total NICU patient days and approximately CA$47 million in total yearly costs [13,14]. The composite outcome of BPD or mortality in EPTs admitted to BCWH has remained high at 61% between 2015 and 2024, with 5% of discharged infants with BPD requiring home ventilation [5,15]. Over 300 clinicians from multiple disciplines deliver interprofessional care; while this leverages complementary expertise, it also contributes to substantial practice variability. A local audit completed in 2023 (unpublished) identified that, while EPTs received more standardized care in the first week of life, the greatest inconsistency occurred in the subsequent six weeks—a period characterized by complex and interdependent decisions related to ventilation, medications, and nutrition.

### 1.3. Limitations of Prior BPD Prevention Interventions

Most prior reported BPD prevention efforts [16,17] have focused on identifying clinical interventions (“what to do”) rather than ensuring their consistent application in practice (“how to do it”) [18,19]. Clinicians have underutilized implementation strategies, which are deliberate evidence-informed methods to improve the adoption, fidelity, and sustainability of clinical practices. Previous approaches have rarely incorporated behavioural or contextual adaptation, team facilitation, or strategies to support decision-making in areas with low- or moderate-certainty evidence. As a result, clinicians have often defaulted to established routines rather than applying emerging evidence tailored to individual infants. These limitations have contributed to inconsistent uptake, low fidelity, and poor scalability [20].

### 1.4. Study Objectives

This study evaluates the levels of reach, effectiveness, adoption, implementation, and sustainment of a multi-component implementation strategy to promote the uptake of clinical interventions to prevent BPD after six months of real-world use.

#### Hypothesis

We hypothesized that a tailored, multi-component implementation strategy for BPD prevention would achieve measurable reach, effectiveness, adoption, implementation fidelity, and early sustainability of clinical interventions within six months of implementation. We further hypothesized that implementation sciences’ best practices, theory- and evidence-based implementation strategies tailored to the specific characteristics of a unit, with high implementation fidelity, would support improvements in teamwork, infant-centred care, and care coordination [21,22]. Sustained, high-fidelity implementation may contribute over time to reductions in the incidence and severity of BPD or rates of BPD-related mortality at 36 weeks’ PMA.

## 2. Materials and Methods

### 2.1. Study Setting

We conducted the study in the NICU at BCWH. BCWH is co-located with BC Children’s Hospital and serves as the provincial referral centre for high-risk perinatal care, extremely preterm infants, major neonatal surgery, and complex neonatal care. The 60-bed unit admits approximately 70 inborn and outborn EPTs annually.

#### 2.1.1. Baseline Team Structure and Continuity of Care

Pre-implementation, care was provided by one of two multidisciplinary teams, each led by a neonatologist. Teams conducted daily morning rounds to identify clinical issues and establish care plans for the subsequent 24 h [23]. A neonatal fellow or hospitalist pediatrician led evening rounds, with participation from the bedside nurse and respiratory therapist. Following the rounds, they sought input from the on-call neonatologist by phone. The team implemented and adjusted clinical interventions based on the evolving patient status. Neonatologists typically provided two consecutive weeks of service to support continuity with off-service neonatologists usually providing on-call weeknight and weekend coverage. Neonatal fellows worked with the same team for at least three days per week, but other multidisciplinary team members had variable continuity across shifts.

#### 2.1.2. Baseline Process for Implementing Clinical Interventions

When new evidence was identified (practice guidelines, systematic reviews, or research), a medical lead and clinical nurse specialist were assigned to develop a local guideline. The neonatal leadership committee reviewed its content and confirmed its local applicability before posting the guideline on the hospital intranet. Providers received the guidelines through educational sessions, expecting clinicians would adopt these guidelines in practice. However, formal guidelines were not developed for interventions supported by low- or moderate-certainty evidence. Clinicians decided individually whether to adopt a given practice, with no monitoring of practice uptake, sustainment, or discontinuation. This contributed to variability in care, lack of evidence of effectiveness, and difficulty in decision-making when multiple management options were available for a given infant.

### 2.2. Intervention: A Quality Improvement Initiative to Reduce BPD

In September 2023, the BCWH Neonatal Programme leadership established a BPD Taskforce comprising three neonatologists and three respiratory therapists to accelerate the adoption of evidence-based practices using existing resources. The team collaborated with family advisors, biomedical technicians, dietitians, pharmacists, clinical nurse specialists, medical sub-specialists, and a Quality and Safety Lead on an ad hoc basis. Initial activities included benchmarking with another centre known to have the lowest BPD rates in Canada and conducting a literature review. The team identified the following three key challenges in adopting new practices: (i) inconsistent application of evidence; (ii) absence of tailored implementation strategies; and (iii) limited infrastructure for sustaining change. These challenges reflect the complexity of neonatal care systems, where outcomes arise from interactions among people, processes, and context [24]. Addressing BPD requires interventions that target behavioural, organizational, and contextual barriers through feedback, reflection, and consensus-based care [25,26].

The BPD Taskforce identified best practices [27], reviewed key interventions [28], and validated the use of the National Institute of Child Health and Human Disease (NICHD) 2022 BPD Outcome Estimator [29]. Unit neonatologists and the respiratory therapy leadership collaborated to undertake a multi-component strategy supporting the implementation of infant-personalized interventions to prevent BPD.

#### 2.2.1. Clinical Interventions and Design of Implementation Strategies

Clinical interventions with potential impact on BPD in EPT care appear in Table S1. The implementation strategies were drawn from established frameworks [21,22,30,31,32,33] and mapped to the priority barriers to implementation that were identified within the local context. The following two systemic postnatal systemic steroid (sPNS) regimens were in use in our unit: (i) Dexamethasone: A Randomized Trial (DART), a tapering 10-day course of intravenous or enteral dexamethasone with a cumulative dose of 0.89 mg/kg [34]; and (ii) Systemic Hydrocortisone to Prevent BPD, a tapering 22-day course with a total cumulative dose of 72.5 mg/kg [35]. Selective early medical treatment of the patent ductus arteriosus in extremely low gestational age infants: a pilot randomized controlled trial protocol (SMART-PDA) [36] was used to treat patent ductus arteriosus. We used the AIMD (aims, ingredients, mechanisms, and delivery) framework [32] to operationalize implementation strategies, and the RE-AIM (reach, effectiveness, adoption, implementation, and maintenance) framework [33] to guide the evaluation (Table S2).

The strategy reframed high rates of BPD and BPD-related mortality as a system issue, requiring process redesign rather than new clinical interventions. It engaged frontline staff and leadership through structured facilitation and feedback, supported training in teamwork and decision-making, enabled data-driven improvement, and promoted sustainability through a community of practice.

#### 2.2.2. Multi-Component Implementation Strategy

The multi-component implementation strategy comprised coordinated approaches to support consistent adoption of clinical interventions to prevent BPD (Table S3). Local guidelines were made readily accessible and usable at the point-of-care to reduce practice variability. High-risk infants were identified using standardized clinical data and validated risk stratification tools. Specifically, the respiratory therapist educator identified all infants less than 29 weeks’ gestation using the electronic health record, input each infant’s variables into the NICHD 2022 BPD Outcome Estimator online tool, and generated the estimated risks of Grade 1, 2, and 3 BPD as well as mortality. Using this information, the respiratory therapist educator created a weekly estimated BPD risk report every Wednesday and shared it with the attending neonatologists. The attending neonatologists prioritized one infant for LCPR every Friday, based on the highest combined estimated risk of Grade 2 or 3 BPD and mortality, as well as clinical management challenges.

Weekly interdisciplinary longitudinal care planning rounds (LCPRs) were conducted to facilitate collaborative decision-making. We structured these one-hour rounds to start with an introduction, where we verbalized Grade 2/3 BPD or mortality risk. Then, we reviewed the respiratory course and potential factors contributing to evolving BPD. Finally, we elicited input from participants on actions to change the illness trajectory (Table S4). Care plans generated during these rounds were documented in a structured, time-bound format within the electronic health record (EHR) to guide execution over the subsequent seven days. Separate dedicated family meetings were convened when complex decisions arose, ensuring clear communication and alignment of care goals between the care team and families.

**Figure 1:**
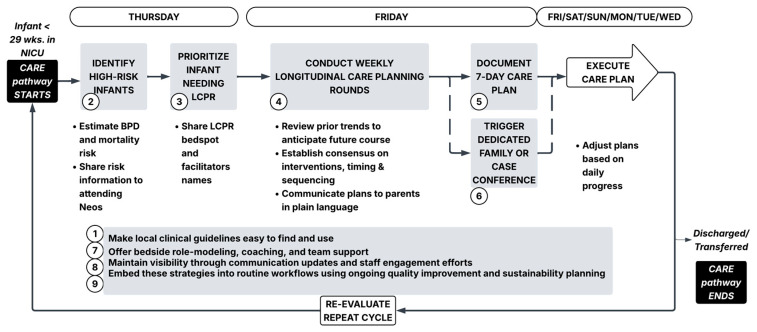
Implementation strategies organized as a care pathway into NICU weekly standard work: numbers 1–9 indicate specific implementation strategies. Abbreviations: NICU, neonatal intensive care; BPD, bronchopulmonary dysplasia; LCPR, longitudinal care planning rounds. Solid arrows represent required steps in the care pathway, while dashed arrows show optional steps that clinicians may undertake when clinically appropriate.

BPD Taskforce members and LCPR facilitators provided ongoing coaching and team support [37,38] while participating in routine clinical care. Regular communication updates and staff outreach reinforced engagement. We integrated these strategies into routine workflows and supported them through ongoing quality improvement processes to ensure sustainability. Figure 1 illustrates implementation strategies structured as a care pathway [39,40].

### 2.3. Study Design and Eligibility

This single-centre prospective mixed-method (qualitative and quantitative) observational implementation study was conducted in a quaternary NICU between October 2024 and April 2025. The study population included all inborn and outborn infants with gestational age <29 weeks admitted to the NICU at <35 weeks’ PMA; it also included healthcare providers involved in these infants’ care (physicians, nurses, respiratory therapists, and allied health staff) and the infants’ parents. We excluded infants if they were outborn and admitted at ≤35 weeks’ PMA, died at ≤7 days of life, were readmitted for non-respiratory surgical issues, or if they had congenital lung anomalies or cyanotic heart disease.

### 2.4. Rollout/Implementation

The pre-implementation phase (May–September 2024) focused on securing approval from Neonatal Program leadership, engaging multidisciplinary team members, and integrating implementation strategies into the unit’s workflow.

The BPD Taskforce led the implementation phase (October 2024–April 2025). In the initial weeks, the team identified operational barriers and refined workflows. Over the first two months, the task force developed and disseminated educational materials, clinical decision algorithms (including postnatal steroid use and extubation readiness), and practice briefs (including inhaled steroids, transpyloric feeding, and non-invasive ventilation with neurally adjusted ventilatory assist [NIV-NAVA]) to physicians and respiratory therapists.

The Taskforce also designed standardized templates for LCPRs, including briefing tools (Figure S1, Files S1 and S2), risk screening reports, structured documentation, and facilitator feedback forms (File S3). We defined standard work by clarifying the roles of neonatologist facilitators, respiratory therapist educators, and bedside providers. Summary plans were posted in the patient’s EHR (see File S4 for example). In parallel, we established methods to assess documentation quality and evaluation.

To support adoption, the Taskforce delivered structured orientation sessions, integrated a standardized toolkit into practice, and provided ongoing bedside coaching and role modelling. We also embedded decision-support resources, including critically appraised topics CATs), locally adopted clinical guidelines, and point-of-care job aids onto routine clinical workflows [41,42]. The sustainment phase (May–November 2025) focused on maintaining these processes within routine practice. Our efforts focused on maintaining strategy use, ensuring tool accessibility via the hospital intranet, disseminating progress though educational and administrative forums, expanding facilitator roles to additional staff, analyzing results, and conducting an end-of-study survey.

### 2.5. Measures and Outcomes

The primary outcome was adoption of the clinical intervention components, evaluated using the RE-AIM framework (Table S3) [35]. The BPD Taskforce conceptualized teamwork and care coordination as proximal outcomes and mechanisms. These mechanisms influence clinical decision-making through structured processes. We evaluated intervention delivery at the following two levels: (i) the infant level; and (ii) the infant-week level. At the infant level, we reported the number of unique EPTs screened and the number who received at least one LCPR during their NICU stay. At the process level, we quantified total screening opportunities and LCPR delivery using infant-weeks as the unit of analysis. An infant-week was defined as one week during which an eligible infant remained in the NICU and met criteria for screening or LCPR consideration. This approach allowed us to assess fidelity of implementation by calculating the proportion of eligible infant-weeks during which LCPR was conducted. We classified care plans from LCPR as high quality if they included: (i) management uncertainty; (ii) decisions required; (iii) defined goals; and (iv) action plans specifying interventions, sequencing, thresholds, and contingencies. Secondary outcomes included exploratory signals related to severity of respiratory illness and duration of respiratory support.

### 2.6. Consent, Ethics and Approval

The institutional research ethics board approved the study as quality improvement under the Tri-Council Policy Statement: Ethical Conduct for Research Involving Humans—TCPS 2 Article 2.5 [43] and waived the requirement for individual consent. Existing approvals permitted use of the Neonatal Program’s discharge database and EHRs. We obtained permission from parents to share the survey link via the Qualtrics platform (Qualtrics software, Version XM [44]), and completion of the survey implied consent; providers gave consent through voluntary participation.

### 2.7. Data Collection

Data sources included EHRs, discharge databases, and patient safety reporting systems. Attendance logs, surveys, chart audits, and implementation team records helped assess implementation processes and outcomes. Observations and meeting notes informed iterative refinement and provided a contextual understanding of implementation successes and barriers.

### 2.8. Sample Size

Prior census data indicated that approximately 35 infants would be eligible during the 7-month study period. We used a convenience sample, given the pragmatic, feasibility-focused study design.

### 2.9. Statistical Analysis

We summarized quantitative data using descriptive statistics. Study team members (A.R.S. and S.S.) reviewed and summarized free-text responses from surveys using conventional content analysis [45]. We performed segmented mixed-effect modelling using R (version 4.3.2) in the RStudio (version 2025.09.1+401) environment [46,47] to assess infant respiratory severity score (RSS) trajectories relative to first discussion on LCPR.

### 2.10. Safety Considerations

The study posed minimal risk. We monitored adverse events and potential overuse of interventions, including postnatal steroids.

## 3. Results

### 3.1. Study Demographics and Interventions Received

**Figure 2:**
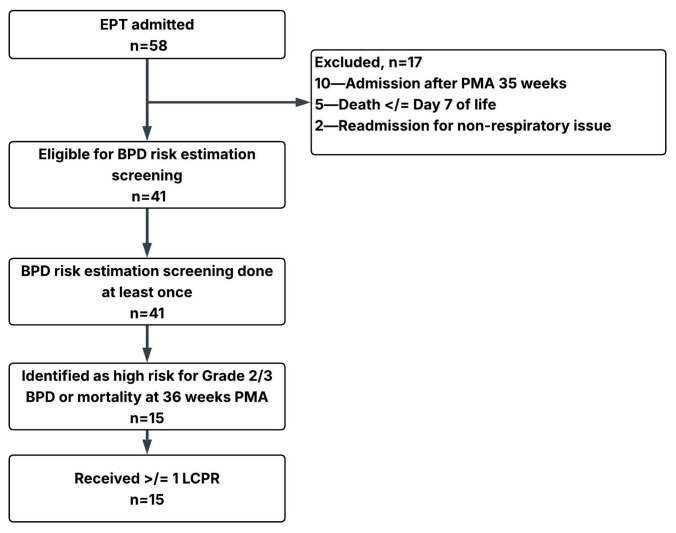
Infant selection and infant-level implementation strategy exposure: counts represent individual infants and reflect intervention reach and uptake. Abbreviations: EPT, extreme preterm; PMA, postmenstrual age; BPD, bronchopulmonary dysplasia; LCPR, longitudinal care planning rounds.

Forty-one infants <29 weeks’ gestational age were part of the study (Figure 2). The median gestational age was 25 weeks (IQR 23–27), with 42% born at 22–24 weeks, 22% at 25–26 weeks, and 37% at 27–28 weeks. Median birth weight was 762 g (IQR 654–975). Female infants comprised 51% of the cohort, and 34% were outborn, representative of the background demographics of our preterm NICU population. All physicians (n = 35), 25 respiratory therapists (62%), two dieticians, three pharmacists, 10 charge nurses (71%) and 20 frontline nurses (8%) took part in the study. Antenatal steroids were administered in 85% of infants, suspected chorioamnionitis was documented in 15%, and 68% were delivered by Cesarean section. At birth, 54% received intubation and mechanical ventilation during resuscitation. Median 5 min Apgar score was 6 (IQR 5–8).

The estimated risk of Grade 2 BPD (nasal cannula > 2 L/min irrespective of FiO_2_, or continuous positive airway pressure [CPAP]/bi-level positive airway pressure [BiPAP]/non-invasive positive pressure ventilation [NIPPV]/non-invasive high-frequency ventilation [NIHFV] irrespective of FiO_2_), or Grade 3 BPD (invasive mechanical ventilation irrespective of FiO_2_) or death per NICHD 2022 BPD Outcome Estimator decreased with advancing postnatal age in the first four weeks of life. The median risks were 37% (IQR 14–54) on day of life (DOL) 1, 29% (11–51) on DOL 3, 22% (11–54) on DOL 7, 19% (15–20) on DOL 14, and 16% (12–22) on DOL 28. The median of the highest estimated risk across time points was 33% (14–63), and the median risk across all time points was 22% (11–50). At any time point, 37% of the infants had a risk of Grade 2/3 BPD or death greater than 50%.

With respect to clinical interventions at any time during admission, 61% of infants received high-frequency ventilation and 78% received surfactant, with 20% receiving more than one dose. Infants received parenteral nutrition for a median of 22 days (IQR 10–37). Hemodynamic and respiratory support included inotropes (dobutamine, epinephrine, or norepinephrine) in 37% and inhaled nitric oxide in 10% of infants. Thirty-seven percent of infants received narcotic infusions (fentanyl, morphine), 22% received sedatives (midazolam, dexmedetomidine), and 10% received muscle relaxants (rocuronium).

Clinicians prescribed sPNS with the aim of decreasing BPD (by facilitating extubation in ventilator-dependent EPTs) to 34% of infants, starting at a median age of 16 days (IQR 13–21) and continuing for a median of 10 days (IQR 10–16). All infants prescribed sPNS received dexamethasone; 14% subsequently received hydrocortisone immediately after dexamethasone to reduce the risk of reintubation related to waning anti-inflammatory effect. On 20% of occasions, clinicians increased the dexamethasone treatment duration to 14 days to ensure successful extubation. Additional respiratory and feeding-related interventions include inhaled steroids (17%), transpyloric feeds (22%) and NIV-NAVA in 27% of infants.

### 3.2. Infant Characteristics and Interventions by LCPR Status

Of the 41 infants in the study, 15 (37%) underwent LCPR (Figure 2). These infants were significantly more premature and smaller compared to those who did not undergo LCPR, with a median gestational age of 24 vs. 27 weeks (*p* = 0.002) and birth weight of 690 g vs. 938 g (*p* = 0.003) (Table 1). They also had higher illness severity, reflected by higher SNAPPE-II scores (53 vs. 42.5, *p* = 0.050), lower 5 min Apgar scores (6 vs. 7, *p* = 0.020), and a higher need for intubation in the delivery room (80% vs. 38.5%, *p* = 0.02). Consistently, estimated risk of Grade 2/3 BPD or death was markedly higher in the LCPR group across all time points, including DOL 1 (53.8% vs. 14.9%, *p* < 0.001), with 73.3% having risk >50% at any time point compared to 15.4% in the non-LCPR group (*p* < 0.001).

Infants in the LCPR group received more intensive interventions, including universal use of high-frequency ventilation (100% vs. 38.5%, *p* < 0.001) and higher exposure to surfactant (100% vs. 65.4%, *p* = 0.010), narcotics (73.3% vs. 15.3%, *p* < 0.001), sedatives (53.3% vs. 3.8%, *p* < 0.001), and inotropes (60% vs. 23.1%, *p* = 0.040). They also had longer duration of parenteral nutrition (40 vs. 11 days, *p* < 0.001) and were more likely to receive systemic postnatal steroids (66.6% vs. 15.4%, *p* = 0.001), including multiple courses (20% vs. 0%, *p* = 0.04). Use of adjunctive strategies such as transpyloric feeding (40% vs. 11.5%, *p* = 0.050) and NIV-NAVA (46.7% vs. 15.4%, *p* = 0.060) was also higher in the LCPR group. Overall, infants selected for LCPR represented a higher-risk, more clinically complex population requiring a greater intensity of care.

### 3.3. Implementation Exposure and Fidelity

Of the EPTs admitted during the study period, all who met eligibility criteria underwent BPD risk estimation screening at least once. We identified a subset of infants as high risk for Grade 2/3 BPD or mortality, and all of these infants received LCPR at least once.

**Figure 3:**
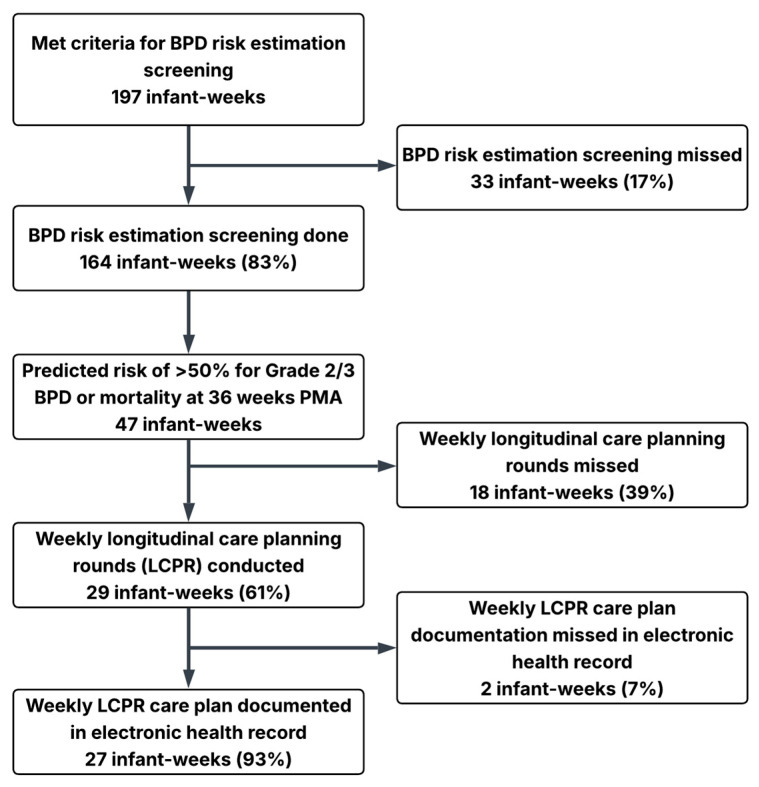
Implementation fidelity using infant-weeks as the unit of analysis (process-fidelity diagram): an infant-week represents one eligible week during which an infant met criteria for screening for LCPR consideration; this analysis quantifies screening intensity and consistency of LCPR delivery over time. Abbreviations: LCPR, longitudinal care planning rounds; BPD, bronchopulmonary dysplasia; PMA, postmenstrual age.

Assessing process fidelity using infant-weeks as the unit of analysis captured the intensity of screening and continuity of intervention delivery. Screening was consistent across eligible time periods, delivery of LCPR was sustained across weeks with infants meeting criteria for consideration, and care plans were documented in nearly all cases (Figure 3).

### 3.4. RE-AIM Evaluation of Implementation

Implementation strategies demonstrated high reach, with 90% of providers exposed through education sessions and all neonatologists receiving weekly risk screening reports (Table 2). Multidisciplinary participation in LCPRs was high among provider groups, though bedside nurses had lower involvement in weekly care planning, and parent participation was dependent on their presence in the NICU.

Effectiveness measures suggested improved standardization of practice, with consistent use of dexamethasone for the first course of sPNS, a median duration of 10 days, and initiation occurring at a median of 16 DOL. We observed the selective use of interventions with low- or moderate-certainty evidence with group consensus, including transpyloric feeding, inhaled steroids, and NIV-NAVA. LCPR also supported the identification of cases that could benefit from the involvement of clinical ethics specialists and structured family discussions.

Adoption of LCPR was supported by consistent provider participation, with involvement of both respiratory therapist educators and most neonatologists as co-facilitators on at least one occasion. Family presence occurred in a subset of rounds, and all identified complex situations resulted in subsequent structured care discussions. Implementation fidelity was high, with most weeks including dissemination of risk screening reports and LCPR followed by structure documentation in the EHR. The care plans contained all predefined essential elements (management uncertainty issues, conceptual goals, and care plans), and the team completed planned actions within the first 3 days (median [IQR] 100% [50–100]), though executing contingency plans proved more variable (75% [50–100]). Reported barriers included time constraints, communication gaps, changes in patient status, and variability in adherence, while facilitators included structured care plans, shared understanding, and strong team engagement.

In surveys (Table 3), respondents valued LCPR for its ability to create a shared mental model, improve team alignment, and support coordinated decision-making in complex cases. Suggested improvements focused on shorter, more goal-directed rounds, stronger facilitation, and better engagement of bedside nurses. Overall, participants viewed LCPR as a collaborative and effective process that improved care planning and team cohesion, with support for continuation alongside refinements in efficiency and implementation.

### 3.5. Exploratory Clinical Outcomes by LCPR Status

Clinical outcomes were broadly similar between infants who received LCPR and those who did not, despite baseline differences in risk (Table 4). Rates of mortality by 36 weeks PMA and the composite outcome of mortality or Grade 2/3 BPD were comparable between groups, with no significant differences observed. Among survivors, the distribution of BPD severity was also similar, with no difference in rates of higher-grade BPD.

Infants in the LCPR group had higher rates of specific complications and interventions, including retinopathy of prematurity requiring treatment, and treatment of patent ductus arteriosus. Other major morbidities, including necrotizing enterocolitis ≥ Stage 2, severe intraventricular hemorrhage, sepsis, periventricular leukomalacia, pneumothorax, and spontaneous intestinal perforation, did not differ significantly between the groups.

Markers of illness severity and resource use were higher in the LCPR group, including longer length of stay, greater duration of invasive ventilation, and increased oxygen exposure. These infants were also more likely to be discharged with gastrostomy tubes and on home non-invasive ventilation. Overall, on unadjusted analysis infants receiving LCPR represented a high-risk population with greater resource utilization, while achieving comparable survival and BPD outcomes to lower-risk infants.

#### Respiratory Severity Score Trajectory

**Figure 4:**
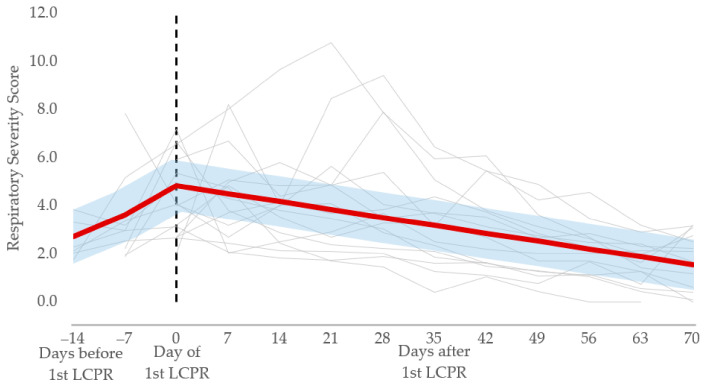
Respiratory severity score (RSS) trajectories relative to the first discussion on longitudinal care planning rounds (LCPRs): on Y-axis, red bold horizontal line represents median (IQR) RSS of all infants, shaded area represents the 95% CI, and faint grey lines represent individual infant trajectories; on X-axis, interrupted black vertical line represents the day of first LCPR for each infant; segmented linear mixed-effect model estimates suggested a downward post-intervention trend (*p* = 0.07).

Across all infants receiving LCPR, there was an overall trend of worsening RSS leading up to the day of first LCPR discussion (day 0) and a subsequent gradual improvement (Figure 4). While individual infant trajectories varied, the overall trend suggested improvement in respiratory status following LCPR. Segmented mixed-effect modelling showed a downward post-intervention trajectory; however, this did not reach statistical significance (*p* = 0.07). These findings suggest a potential, but not definitive, association between LCPR and improvement in respiratory severity.

## 4. Discussion

### 4.1. Implementation Outcomes and Practice Standardization

After six months, the multi-component implementation strategy demonstrated strong reach, effectiveness, adoption, and implementation fidelity, with sustained integration into routine clinical practice despite some variability in execution. We integrated the implementation strategies within NICU workflows without adversely affecting patient outcomes. Preliminary indicators demonstrated a possible improvement in respiratory severity after implementation. Importantly, high-risk infants attained survival rates and BPD outcomes comparable to those observed in the lower-risk group. Providers also reported improvements in teamwork, team culture, care coordination, self-efficacy, and confidence.

The high levels of reach and adoption observed in this study were likely driven by structured education, consistent dissemination of weekly risk screening reports, and active engagement of a multidisciplinary team, including regular co-facilitation by neonatologists and respiratory therapist educators. Systematic risk stratification enabled targeted identification of high-risk infants, ensuring that LCPR was applied to those most likely to benefit and enhancing both clinical relevance and uptake. Implementation fidelity was supported by standardized documentation, clearly defined care plans with contingency strategies, and integration into routine workflows, although variability in execution highlighted ongoing challenges related to communication and consistency. The intervention also appeared to enhance effectiveness by improving team alignment, promoting standardized practices such as steroid use, and facilitating structured decision-making in complex clinical and ethical scenarios. Sustained maintenance was likely enabled by continued use beyond the study period, expansion of facilitator roles, strong team engagement, and ready access to implementation tools, supporting normalization into routine care. The observed trend toward improvement in respiratory severity scores may reflect the impact of more standardized and coordinated personalized care, consistent with prior reports [48,49,50].

Compared with a prior cohort at our centre (2011–2021) [30], a higher proportion of infants in the current study received sPNS (34% vs. 21%), while fewer required multiple courses (7% vs. 39%). Initiation occurred earlier (median [IQR] 16 [12,13,14,15,16,17,18,19,20,21] vs. 29 [19,20,21,22,23,24,25,26,27,28,29,30,31,32,33,34,35,36,37,38] days), and clinicians consistently used dexamethasone as the initial course (100% vs. 85%). These differences likely reflect improved identification of infants at risk for Grade 2/3 BPD through systematic risk stratification, enabling earlier, team-based consensus on initiating a standardized dexamethasone regimen. More effective and sustained extubation may also have reduced the need for repeated steroid exposure. Compared to a more recent cohort at our centre (<29 weeks’ gestation at birth, 2022–23) [30], BPD diagnosis at 36 weeks’ PMA among survivors was higher (81% vs. 46%), but the unadjusted mortality rate was lower (7% vs. 13%), and fewer infants were discharged home on mechanical ventilation (5% vs. 8%). However, the current study cohort were younger (median [IQR] weeks’ gestation at birth 25 [23,24,25,26,27] vs. 26 [25,26,27,28]) and sicker (median [IQR] SNAPPE-II score 46 [31,32,33,34,35,36,37,38,39,40,41,42,43,44,45,46,47,48,49,50,51,52,53] vs. 27 [18,19,20,21,22,23,24,25,26,27,28,29,30,31,32,33,34,35,36,37,38,39,40,41,42]). We speculate that the higher BPD rate observed in the current study may stem from improved survival in a higher-risk population.

### 4.2. Implementation Challenges and Adaptations

We encountered five key challenges during implementation and addressed each through targeted adaptations. First, aligning BPD risk estimation with fixed NICHD 2022 BPD Outcome Estimator [5,51] time points (DOL 1, 3, 7, 14, and 28) was difficult because our assessments were conducted weekly on Thursdays. To address this, respiratory therapist educators used the infant’s day of life closest to the predefined time points, and for infants beyond DOL 27, day 28 estimates were applied with this limitation clearly documented in weekly reports.

Second, early concerns among staff regarding parental participation in LCPR centred on the potential for increased anxiety and the use of complex, non-plain language during discussions of risk, prognosis, and differing clinical opinions. To address this, we actively sought parent feedback during the initial two months through surveys. Parents consistently expressed a strong preference to be included, aligning with prior studies [52,53,54], and emphasized the need for clear, plain-language summaries. In response, we formally integrated parent participation into LCPR by inviting them and establishing routine provision of structured, plain-language summaries of discussions and care plans.

Third, concerns about loss of autonomy among most responsible physicians (MRPs) were mitigated by preserving their central role in decision-making. MRP neonatologists were involved in identifying eligible infants, invited to present their care plans upfront, and retained the ability to modify LCPR recommendations. This approach maintained clinical ownership while fostering collaborative input and trust.

Fourth, bedside nurse engagement was lower than expected, with only 22 bedside nurses (representing 10%) reporting taking part in LCPR. This may reflect the competing demands placed on nurses, involvement of multiple providers in rounds, and the relatively low likelihood that any individual nurse would regularly care for an infant requiring LCPR. Despite this, we have clarified the specific role of bedside nurses in the structured template that guides LCPR, including their contribution to care requirements, pain and comfort measures, supportive strategies that are working well, and priorities they would like included in the weekly care plan. Including these nursing perspectives helps ensure the LCPR care plan supports not only respiratory management but also continuity of bedside nursing care.

Finally, variability in execution of care plans beyond 72 h was observed. Review showed that much of this variation was clinically appropriate, reflecting changes in patient condition, such as intercurrent illness. In some cases, even when plans were followed, gaps in supportive care contributed to unintended outcomes like reintubation. These findings underscore the need for ongoing reassessment, flexibility, and reinforcement of supportive practices to ensure effective and adaptive implementation of care plans.

### 4.3. Comparison with the Prior Literature

While prior studies have outlined clinical strategies to prevent BPD from the delivery room through the NICU, including ventilatory and pharmacologic approaches [55,56,57,58], few have explicitly examined how these interventions are implemented in practice [59]. A large NICU quality improvement initiative showed that standardization, age-specific protocols combined with consistent team practices, and a shared mental model can reduce BPD rates. Their approach emphasized daily bedside rounds, weekly team meetings, case-based discussions, and consensus-driven decisions [59]. However, that work did not formally evaluate its implementation strategies, did not incorporate parent participation, and lacked risk-based selection of infants for targeted discussions. In contrast, our study extends this approach by systematically evaluating implementation using a structured framework, incorporating parents into care planning, and using BPD risk stratification to direct interventions toward infants most likely to benefit.

Our findings demonstrate that a multi-component implementation strategy to promote the uptake of clinical interventions to prevent BPD can be successfully sustained in a real-world NICU setting. This can lead to meaningful improvements in care processes, team functioning, and practice consistency. However, whether these gains translate into long-term improvements in clinical outcomes such as BPD, mortality, and length of stay remains to be determined and warrants further study. Future work should also evaluate the cost-effectiveness and overall value of adopting this approach. It should evaluate short-term patient-centred outcomes such as severity of BPD, chronic pulmonary hypertension, duration and type of respiratory support, tracheostomy rates, as well as long-term neurodevelopmental and pulmonary morbidities. The strategy appears adaptable and potentially scalable, either as a complete model or through selected components. That said, successful adoption will require centres to account for the reorganization of their resources needed to support ongoing risk assessment, multidisciplinary rounds, and structured documentation.

### 4.4. Strengths and Limitations

This study has several strengths. We applied established implementation frameworks to both the design and evaluation of the intervention, demonstrated sustained integration within a clinical setting, and combined clinical and implementation strategies to support personalized, infant- and family-centred care. The approach also fostered a shared culture of consensus, accountability, and coordinated decision-making.

This study also has important limitations. We conducted the study at a single centre with a relatively small sample size and without a matched comparator group. This limits the ability to determine whether observed trends in respiratory severity or comparable outcomes among high-risk infants were directly due to the intervention. Comparisons between infants who received LCPR and those who did not were unadjusted for differences in demographics or illness severity due to the small sample size. In addition, the use of validated instruments to assess domains such as teamwork, care coordination, psychological safety, self-efficacy, and confidence would be opportunities for future improvements in our approach. The intervention bundle incorporated multiple simultaneous clinical and implementation strategies, including sPNS, NIV-NAVA, transpyloric feeds, facilitation, coaching, and structured documentation. As a result, it is difficult to determine which components contributed most significantly to the observed effects. Thus, we should interpret clinical outcome findings as exploratory and hypothesis-generating within the context of a demonstration project. Unmeasured biological factors, including genetic predisposition and infant-specific disease trajectories, may have influenced the outcomes. These factors may also limit how much we can attribute improvements solely to the intervention. Future studies with larger cohorts and more detailed biological data could explore how genetic susceptibility interacts with care processes in the development of BPD.

## 5. Conclusions

A multi-component implementation strategy to support the delivery of clinical interventions for BPD prevention achieved high reach, effectiveness, adoption, and implementation fidelity, with clear evidence of sustained integration into routine practice. We observed early signals suggesting a potential improvement in respiratory severity following implementation. Notably, high-risk infants achieved survival and BPD outcomes comparable to those of lower-risk infants following implementation of our intervention. Providers also reported improvements in teamwork, team culture, care coordination, self-efficacy, and confidence.

## Figures and Tables

**Table 1:** Baseline characteristics and interventions received comparing infants who received LCPR and those who did not.

Characteristics	LCPR (n = 15)	No LCPR (n = 26)	*p*-Value
Gestational age, weeks, median (IQR)	24 (23–25)	27 (24–28)	0.002
Birth weight, g, median (IQR)	690 (562–736)	938 (706–1106)	0.003
Female	6 (40.0)	15 (57.7)	0.340
Outborn	4 (26.7)	10 (38.5)	0.510
Antenatal steroids (partial or complete)	14 (93.3)	21 (80.8)	0.380
Suspected chorioamnionitis	3 (20.0)	3 (11.5)	0.650
Cesarean delivery	9 (60.0)	19 (73.1)	0.490
Intubation at resuscitation	12 (80.0)	10 (38.5)	0.020
SNAPPE-II, median (IQR)	53 (45.5–56.5)	42.5 (22–50.5)	0.050
Apgar 5 min, median (IQR)	6 (5–7)	7 (6–9)	0.020
**Estimated risk of Grade 2/3 BPD or mortality (%)**			
**Timepoint**	**LCPR**	**No LCPR**	***p*-value**
Day 1, median (IQR)	53.8 (42.8–65.7)	14.9 (9.9–32.7)	<0.001
Day 3, median (IQR)	49.7 (34.3–63.3)	12.6 (7.6–21.0)	<0.001
Day 7, median (IQR)	54.2 (37.7–63.8)	11.3 (9.2–31.5)	<0.001
Day 14, median (IQR)	48.7 (29.6–63.4)	12.2 (8.7–19.2)	<0.001
Day 28, median (IQR)	22.2 (16.2–34.8)	13.5 (9.7–16.4)	0.001
Highest risk in first 28 days, median (IQR)	65.9 (49.6–73.5)	15.5 (12.4–33.8)	<0.001
Risk > 50% at any time point	11 (73.3)	4 (15.4)	<0.001
**Interventions Received**	**LCPR**	**No LCPR**	***p*-value**
Peripheral arterial line	0 (0)	3 (11.5)	—
Parenteral nutrition days, median (IQR)	40 (29.5–66.5)	10.5 (8–24.2)	<0.001
High-frequency ventilation	15 (100.0)	10 (38.5)	<0.001
Surfactant (any)	15 (100.0)	17 (65.4)	0.010
Surfactant >1 dose	3 (20.0)	5 (19.2)	1.000
Narcotic infusion	11 (73.3)	4 (15.4)	<0.001
Sedatives	8 (53.3)	1 (3.8)	<0.001
Muscle relaxants	3 (20.0)	1 (3.8)	0.130
Inhaled nitric oxide	3 (20.0)	1 (3.8)	0.130
Inotropes	9 (60.0)	6 (23.1)	0.040
Any sPNS	10 (66.6)	4 (15.4)	0.001
Dexamethasone as first course of sPNS	10 (66.6)	4 (15.4)	0.001
>1 sPNS course	3 (20.0)	0 (0.0)	0.040
Total sPNS days, median (IQR)	10 (10–21)	10 (9–11)	0.360
Day of first sPNS course, median (IQR)	15 (11–20)	21 (19–25)	0.150
Dexamethasone course followed by immediate hydrocortisone course to prevent reintubation	2 (13.3)	0 (0.0)	
Inhaled steroids	5 (33.3)	2 (7.7)	0.070
Transpyloric feeds	6 (40.0)	3 (11.5)	0.050
NIV-NAVA	7 (46.7)	4 (15.4)	0.060

Each cell represents n (%) unless otherwise specified. BPD grade as defined by Jensen et al. [5]. Abbreviations: LCPR, longitudinal care planning rounds; BPD, bronchopulmonary dysplasia; sPNS, systemic post-natal steroids; NIV-NAVA, non-invasive ventilation with neurally adjusted ventilatory assist.

**Table 2:** Level of reach, effectiveness, adoption, implementation, maintenance, and sustainability (RE-AIM evaluation) metrics.

**Reach**	
Proportion of providers exposed to guidelines and LCPR in education days	179/200 (90%)
Proportion of providers exposed to LCPR during the study period	All respiratory therapists (n = 35), physicians (n = 32), dieticians (n = 4), pharmacists (n = 4), charge nurses (n = 16), managers (n = 2); 22/218 (10%) nurses
Proportion of neonatologists exposed to risk estimate weekly screening report, during the study period	14/14 (100%)
Proportion of parents joining LCPR at least once during the study period	3/15 (20%)
**Effectiveness**	
Trend of weekly median (95% CI) respiratory scores over time for infants who received weekly care planning rounds.	See Figure 4
Perceived teamwork, care coordination, self-efficacy/confidence	See Table 3
Variation in practice: practices during the study period	
Proportion of infants receiving sPNS who had 10-day dexamethasone regimen as 1st course of sPNS;	14/14 (100%);
Total days of sPNS, Med(IQR);	10 (10–15);
Proportion of infants who received >1 course of sPNS.	3/14 (21%).
Timing of initiation of interventions—time to initiation of first course of sPNS, median (IQR) days of life	16 (12–21)
Proportion of infants who received interventions with low or moderate certainty of evidence:	
Transpyloric feeding;	9/41 (22%);
Inhaled steroids;	7/41 (17%);
NIV-NAVA.	11/41 (27%).
Recognize situations where ethical/moral/social issues need structured team and/or family conversations among infants who received LCPR	3/15 (20%)
**Adoption**	
Participation rate/LCPR- Med(IQR)	
All providers;	16 (14–18);
Neonatologists.	5 (3–6).
Proportion of LCPR where parents were present	9/29 (31%)
Proportion of RT educators/instructors who co-facilitated LCPR at least once	4/4 (100%)
Proportion of neonatologists who co-facilitated LCPR at least once	8/14 (57%)
**Implementation**	
Proportion of LCPR where sPNS use according to unit guidelines were discussed	10/29 (34%)
Proportion of weeks when neonatologists or their delegates received the risk-estimation screening report.	24/26 (88%)
Proportion of weeks where LCPR was followed by a documented weekly care plan in the electronic health record	27/29 (93%)
Quality of documented LCPR care plan—proportion of documented care plans in EHR that had essential elements.	26/27 (96%)
Proportion of planned items in LCPR care plan, completed by the care team within a specified time window across all LCPR occasions, Med (IQR)	
≤3 days;	100% (50–100);
≤7 days.	75% (50–100).
Proportion of suggested contingency actions executed per LCPR applicable occasion within 7 days by the care team across all LCPR occasions, Med (IQR)	38% (31–50)
Barriers: time burden, poor visibility/awareness of care plans, inconsistent adherence over time, unpredictable patient changes derailing execution, communication gaps between providers, provider variability and differing opinions, delay in facilitators signing-up, lack of outcome data	
Facilitators: clear structured care plans, shared mental model, accessible documentation, contingency planning, consistency across handovers, strong team buy-in, effective facilitation, perceived clinical benefit	
**Maintenance**	
Proportion of weeks when neonatologists or their delegates received the risk-estimation screening report in the six months beyond the study period	26/26 (100%)
Proportion of weeks where LCPR occurred in the six months beyond the study period	21/26 (81%)
Proportion of clinicians reporting participation in LCPR at least once in the six months beyond the study period	45/62 (73%)
Proportion of neonatologists who co-facilitated LCPR at least once	14/14 (100%)
Proportion of clinicians reporting the spread of study interventions and strategies beyond the target population	34/44 (77%)
Study toolkit posted on hospital intranet for easy access	Yes

Abbreviations: LCPR, longitudinal care planning rounds; sPNS, systemic postnatal steroids; NIV-NAVA, non-invasive ventilation with neurally adjusted ventilatory assist; RT, respiratory therapist; EHR, electronic health record.

**Table 3:** Perceived impact of longitudinal care planning rounds (LCPRs), documentation, and care plans by domain (agree or strongly agree responses among survey responders).

Domains and Statements	n/N (%)
**1. Teamwork and team culture**	
Enhanced shared decision-making with the care team	41/51 (80%)
Improved interprofessional collaboration and communication	42/51 (82%)
Fostered psychological safety and supportive environment	39/51 (77%)
Encouraged reflection and shared team learning	42/50 (84%)
Strengthened shared accountability and trust	38/46 (83%)
Improved overall teamwork and communication	36/45 (80%)
Strengthened shared learning and collaboration	38/45 (84%)
*Domain summary (median % agreement across items):*	*82%*
**2. Care Coordination and Reliability**	
Improved coordination of care for high-risk infants	44/51 (86%)
Improved sequencing of interventions	40/50 (80%)
Supported contingency planning	40/49 (81%)
Enabled time-limited trials	34/48 (70%)
Reduced variability and promoted consistent care	36/49 (73%)
Documentation improved continuity and clarity of care	35/46 (76%)
Documentation standardized communication during handovers	37/46 (80%)
Care plans consistently followed	27/46 (59%)
Reduced variation and improved reliability of care (overall)	34/45 (76%)
Weekly risk assessment helped identify high-risk infants	38/44 (86%)
*Domain summary (median % agreement across items):*	*80%*
**3. Self-Efficacy and Confidence**	
Improved confidence in managing high-risk infants	36/51 (71%)
Increased confidence in evidence-based decisions	31/45 (69%)
Improved ability to apply evidence-based practices to other infants	40/51 (78%)
Confident implementing and adjusting care plans	27/45 (60%)
Applied learning from LCPR to other infants	34/44 (77%)
*Domain summary (median % agreement across items):*	*71%*

**Table 4:** Outcomes comparing infants who received LCPR vs. those who did not.

Characteristics	LCPR (n = 15)	No LCPR (n = 26)	*p*-Value
Mortality (anytime)	1 (7.7)	2 (7.4)	1.000
Mortality by 36 weeks PMA	0	1 (3.8)	1.000
Mortality or Grade 2/3 BPD at 36 weeks PMA	12 (80.0)	19 (73.1)	0.710
BPD grade ^1^ at 36 weeks PMA among survivors			
Grade 1/2/3	13 (86.6)	19 (73.1)	0.470
Grade 2	12 (80.0)	18 (69.2)	0.510
Grade 3	0 (0)	1 (3.8)	1.000
ROP-treated	7 (50.0)	2 (8.3)	0.006
PDA-treated	11 (73.3)	8 (30.8)	0.010
NEC ≥ Stage 2	2 (13.3)	2 (7.7)	0.610
IVH ≥ Grade 3	5 (33.3)	6 (23.1)	0.410
Culture positive sepsis	7 (46.7)	8 (30.7)	0.330
Spontaneous intestinal perforation	0 (0.0)	2 (7.7)	0.520
PVL	2 (13.3)	2 (7.7)	0.610
Pneumothorax	1 (6.7)	2 (7.7)	1.000
Discharged with gastrostomy tube	4 (28.6)	0 (0.0)	0.001
Discharged home on non-invasive ventilation	2 (14.3)	0 (0.0)	0.040
Survival to discharge or transfer without major morbidity ^2^	2 (14.3)	6 (25.0)	0.680
Length of stay (days), median (IQR)	124 (93–219)	65 (49–95.5)	<0.001
Invasive ventilation days, median (IQR)	18 (13–34)	2 (0–10.3)	<0.001
Oxygen days, ^3^ median (IQR)	80 (60–185)	55 (45–82.8)	0.010

Each cell represents n (%) unless otherwise specified. Abbreviations: PMA, postmenstrual age; BPD, bronchopulmonary dysplasia; ROP, retinopathy of prematurity; PDA, patent ductus arteriosus; NEC, necrotizing enterocolitis; IVH, intraventricular hemorrhage; PVL, periventricular leukomalacia. Footnotes: ^1^ as defined by Jensen et al. [5]; ^2^ survival without NEC stage ≥ 2. ROP stage ≥ 3, PVL or IVH grade ≥ 3 at discharge, or BPD grade 2/3 at 36 weeks’ PMA; ^3^ estimated as duration between first and last day receiving supplemental oxygen.

## Data Availability

The datasets presented in this article are not readily available because they consist of de-identified patient data collected for local quality improvement purposes and are subject to institutional privacy and data governance restrictions. Requests to access the data should be directed to the corresponding author and will be subject to institutional review and approval.

## Supplementary Materials

The following supporting information can be downloaded at https://www.mdpi.com/article/10.3390/jpm16060303/s1, Table S1: Neonatal Clinical Interventions Relevant to BPD Prevention in EPT Care; Table S2: Evaluation Plan; Table S3: Theory-driven Implementation Plan using AMID Framework; Table S4: LCPR Structured Template; Figure S1: Decision Algorithm for Initiating First Course of sPNS; File S1: Evidence Brief and Recommendations on Inhaled Steroids; File S2: Evidence Brief and Recommendations on Transpyloric Feeding in EPTs on Ventilatory Support; File S3: Facilitator Feedback Form. File S4. Example of documented LCPR summary plan in electronic health record.

## Abbreviations

The following abbreviations are used in this manuscript:

EPT	Extreme preterm
BPD	Bronchopulmonary dysplasia
PMA	Postmenstrual age
BCWH	BC Women’s Hospital + Health Centre
NICHD	National Institute of Child Health and Human Disease
AIMDs	Aim, ingredients, mechanism, and delivery
RE-AIM	Reach, effectiveness, adoption, implementation, and maintenance
LCPRs	Longitudinal care planning rounds
EHR	Electronic health record
NIV-NAVA	Non-invasive ventilation with neurally adjusted ventilatory assist
CAT	Critically appraised topic
RSS	Respiratory severity score
DOL	Day of life
sPNSs	Systemic postnatal steroids
MRP	Most responsible provider
