# Supplementary material for: Risk-Guided Personalized Care to Prevent Bronchopulmonary Dysplasia: A Real-World Implementation Study
Source: J Pers Med. 2026 Jun 3;16(6):303. doi: 10.3390/jpm16060303 (PMC13301073; doi:10.3390/jpm16060303)
Supplement: Supplementary file 1 [file jpm-16-00303-s001.zip › jpm-4285526-supplementary.pdf]

Table S1. Neonatal Clinical Interventions Relevant to BPD Prevention in EPT Care

**Domain**	**Description**	**Examples of Interventions**
1. Respiratory Support Interventions	Interventions influencing lung injury, oxygen exposure, and ventilator-induced damage	Mode of ventilation (CMV, HFOV, HFJV); Non-invasive ventilation (CPAP, NIPPV, NIV-NAVA, HFNC); Oxygen saturation targeting; PEEP strategies; Extubation timing; Reintubation thresholds; Inhaled nitric oxide; Surfactant (repeat dosing); Inhaled steroids; Ventilator weaning strategies; Recruitment maneuvers
2. Pharmacologic Interventions	Medication-based therapies targeting respiratory, cardiovascular, infectious, and sedation pathways	Lung-targeted: Systemic postnatal steroids (dexamethasone, hydrocortisone); Inhaled steroids; Diuretics; Caffeine. Cardiovascular: Inotropes; PDA treatment (indomethacin, ibuprofen, acetaminophen); Pulmonary hypertension therapies (e.g., sildenafil, diuretics). Anti-infective: Antibiotics; Antifungal; Antiviral; Ureaplasma-directed therapy. Sedation/Analgesia: Narcotics; Benzodiazepines; Muscle relaxants
3. Nutritional Interventions	Strategies supporting growth, lung development, and metabolic stability	Protein and calorie targets; Fluid restriction; Human milk vs formula; Fortification strategy; Transpyloric vs gastric feeds; Timing of enteral feed advancement; Feeding intolerance management; Metabolic bone disease prevention
4. Hemodynamic Management	Interventions targeting circulatory stability and oxygen delivery	Blood pressure targets; Volume expansion; PDA management strategy (conservative vs active); Transfusion thresholds; Hemoglobin targets
5. Fluid and Electrolyte Management	Optimization of fluid balance and electrolyte stability	Fluid restriction; Diuretic strategy; Sodium supplementation; Electrolyte correction thresholds
6. Infection Prevention and Management	Strategies reducing infectious morbidity and inflammation	Antibiotic stewardship; Line care practices; Ventilator-associated infection prevention; Early sepsis evaluation strategy
7. Procedural / Device-Based Interventions	Invasive devices and procedural supports impacting respiratory and systemic care	Arterial lines; Central lines; Timing of line removal; Transpyloric tube placement; Gastrostomy tube placement
8. Monitoring and Investigations	Diagnostic and surveillance tools guiding clinical decisions	Blood gases; Chest radiograph frequency; Echocardiography timing; Risk estimation tools (e.g., NICHD calculator); Ventilator parameter trending; Biomarkers (if used)
9. Developmental and Neuroprotective Care	Interventions promoting neurodevelopment and minimizing stress-related injury	Minimal handling; Kangaroo care; Noise/light reduction; Positioning strategies; Early OT/PT involvement; Family-integrated care
10. Escalation / Rescue Strategies	Advanced or rescue interventions for clinical deterioration	Escalation to HFOV; Rescue steroids; Surgical PDA ligation
11. Ethical and Goal-Concordant Care Decisions	Structured approaches to aligning care with family values and prognosis	Goals-of-care discussions; Comfort-focused care decisions; Family case conferences

Abbreviations: EPT, extreme preterm; CMV, continuous mechanical ventilation; NICHD, National Insitute of Child Health and Human Disease; HFOV, high-frequency oscillatory ventilation; HFJV, high-frequency jet ventilation; CPAP, continuous positive airway pressure; NIPPV, non-invasive positive pressure ventilation; NIV-NAVA, non-invasive ventilation with neurally-adjusted ventilatory assist; HFNC, high-flow nasal cannula; PEEP, positive end-expiratory pressure; PDA, patent ductus arteriosus

Table S2. Evaluation Plan

Dimension	Measure(s) and operational definition	Data Source / Method
**Reach-** Absolute number or proportion of individuals involved in or exposed to the implementation strategies.	• Proportion of providers exposed to guidelines and LCPR in education days<br>• Proportion of providers exposed to LCPR during the study period<br>• Proportion of of neonatologists/delegates exposed to risk estimate weekly screening report, during the study period<br>• Proportion of parents joined LCPR at least once during the study period	Weekly risk estimation reports<br>Facilitator evaluation form<br>Unit admission registry<br>Unit staff scheduling registry<br>Email distribution log<br>Survey
**Effectiveness-** The impact of the intervention on important process level outcomes	• Trend of weekly median (IQR) respiratory severity scores [1] over time for infants who received weekly care planning rounds.<br>• Perceived Team work[2], care coordination[3], self-efficacy/confidence[4]<br>• Variation in practice- Choice of first course of sPNS, median (IQR) days of sPNS, courses of PNS<br>• Timing of initiation of interventions- Time to initiation of first course of postnatal systemic steroids (sPNS), Median (IQR)<br>• Use of interventions with low or moderate certainty of evidence, such as transpyloric feeding, inhaled steroids and NIV-NAVA during the study period<br>• Recognise situations where ethical/moral/social issues needed dedicated structured team and/or family conversations among infants who received LCPR [5]	Chart audits from EHR<br>Provider and parent surveys –<br>Historical cohort data
**Adoption-** Uptake by providers.	• Median (IQR) participation rate/ LCPR- (all providers, Neos)<br>• Proportion of LCPR where Parents were present<br>• Proportion of LCPR in which identified situations involving ethical, moral, or social issues led to structured care conferences or discussions with family to elicit and integrate family values in LCPR.<br>• Proportion of RT educators/instructors who co-facilitated LCPR at least once<br>• Proportion of neonatologists who co-facilitated LCPR at least once	Weekly risk estimation reports<br>Facilitator evaluation form<br>Email distribution logs
**Implementation-** The fidelity (extent to which each component is delivered as intended) consistency, and penetration (Proportion of infants / providers / parents receiving implementation strategies) with which the intervention is delivered. (degree of use)	• Proportion of LCPR where sPNS use according to unit guidelines were discussed<br>• Proportion of weeks when neonatologists or their delegates received the risk estimation screening report.<br>• Proportion of weeks where LCPR was followed by a documented weekly care plan in the electronic health record<br>• Quality of documented LCPR care plan- Proportion of documented care plans in EHR, that had essential elements.	Weekly risk estimation reports<br>Facilitator evaluation form<br>Audit of documented weekly care plans in EHR<br>Hospital adverse event reporting database<br>List of barriers and facilitators for multiple strategies

Table S2 (cont.)

**Implementation** (cont.)	• Overall plan execution[6]: Median (IQR) proportion of planned care components (recommended investigations and interventions) completed by the care team within defined timeframes (≤3 days, ≤7 days) across all LCPR encounters.<br>• Overall contingency plan execution: Median (IQR) proportion of recommended contingency actions implemented by the care team within 7 days for each applicable LCPR encounter.<br>List of barriers and facilitators for multiple strategies	
**Maintenance/ sustainability Integration/ normalization.** The extent to which the intervention is sustained over time, multi-component strategies become routine, expected, and embedded in everyday clinical work.	• Proportion of weeks when neonatologists or their delegates received the risk estimation screening report in the six months beyond the study period<br>• Proportion of weeks where LCPR occurred in the six months beyond the study period<br>• Proportion of providers reporting participation in LCPR at least once in the six months beyond the study period<br>• Evidence of spread beyond the target population. Proportion of providers reporting the spread of study interventions and strategies beyond the target population<br>• Study toolkit posted on the intranet for easy access	Policy and workflow documents (observation, team meeting notes)<br>Weekly risk estimation reports<br>Facilitator evaluation form<br>Audit of documented weekly care plans in EHR<br>Provider survey

1. Respiratory severity scores (RSS)= MAP x FiO_2
2. Perceived teamwork: Clinicians' assessment of how effectively NICU team members collaborate during activities to prevent BPD, including communication quality, shared decision-making, role clarity, and mutual support in implementing agreed interventions.
3. Perceived Care coordination: The degree to which care interventions are delivered in a timely, organized, and consistent manner across providers, shifts, and disciplines, ensuring continuity of care aligned with the care bundle.
4. Perceived Self-efficacy / confidence: Clinicians' perceived ability and confidence to apply care practices correctly, make care decisions aligned with the care plan, and address clinical challenges related to BPD prevention and management.
5. Included structured complex care multidisciplinary discussions convened in a meeting room, to address prognostic uncertainty, ethical challenges, and differing family and team goals; *Supporting Infants with Medical Fragility and Ongoing Need for Intensive Care (SIMFONIC) rounds*
6. For each LCPR occasion, we generated a list of **plan items** (investigations, interventions, contingencies). Then we calculated the **percentage of those items completed within the defined time frame (**Denominator: Total number of planned items documented during that LCPR occasion
   Numerator: Number completed within the predefined time window). Finally, we report **the median (IQR) across all LCPR occasions**.

Abbreviations:
LCPR, longitudinal care planning rounds; EHR, electronic health record; sPNS, systemic postnatal steroids; RT, respiratory therapist

Table S3. Theory-driven Implementation Plan Using AIMD Framework

**Barriers to Implementation**	**Implementation strategy**	**Aim of Implementation Strategy (for whom)**	**Key Active Ingredients**	**Mechanism of Action (Intervention Functions)**	**Delivery Mode**
Decision paralysis; inconsistent uptake of low/moderate certainty practices; lack of structured decision-support	Make local clinical practice guidelines accessible	Standardize care and support timely, evidence-informed decisions (care team)	Localized guidelines, rapid briefs, algorithms, time-limited trial runs	Education + Environmental Restructuring: builds knowledge, confidence, and shared norms for timely, consistent decisions	Develop / distribute materials; local consensus; reminders; pocket cards; intranet summaries
Missed identification of high-risk infants needing longitudinal care planning	Perform data-driven risk stratification, audit and feedback with action-oriented reminders	Focus attention/resources on infants most at risk (care team)	Risk stratification using NICHD BPD calculator and Neo/RT joint review	Enablement + Training: strengthens awareness and coordination using data-driven cues	Data-driven feedback via email, in-person, or text
Fragmented or delayed decisions; disengagement, risk-averse culture	Conduct interdisciplinary facilitated structured weekly longitudinal care planning rounds	Ensure coordinated teamwork and reliable execution (care team)	Facilitated rounds; standardized templates; staff recognition	Modeling + Persuasion + Enablement: fosters collaboration, accountability, and motivation	Weekly rounds; coaching; feedback loops; identify champions
Ethical/social/moral dilemmas; family stress	Trigger structured family values clarification meetings or complex care conferences*	Align care with family values and reduce moral distress (care team and families)	Screening questions, dialogue guides, family inclusion, ethics framing	Persuasion + Education + Enablement: encourages open dialogue, value alignment, and conflict reduction	Structured family meetings or referral to the existing complex care conference meetings (virtual/in-person)
Limited engagement or inconsistent uptake among staff	Provide role modelling, coaching and implementation team support	Build collective ownership and readiness for implementation (care team)	Multidisciplinary implementation team; bedside role modeling and coaching	Modeling + Enablement: promotes peer learning, engagement, and local ownership	Orientation, bedside mentorship, and coaching, surveys,
Low visibility; competing priorities; limited feedback	Use communication and visibility campaign to	Maintain awareness and motivation to engage (all staff)	Posters, newsletters, communication champions	Persuasion + Incentivization + Reinforcement.	Newsletters, lunch and learn meetings

	maintain staff engagement			reinforces motivation through recognition and shared progress	
Implementation fatigue; lack of long-term integration into policy and practice	Integrate strategies into unit's standard workflow using iterative quality improvement cycles and sustainability planning	Sustain motivation and continuous learning (care team)	Iterative refinement; feedback loops; toolkit and policy updates	Enablement + Environmental Restructuring + Incentivization: supports adaptability and embeds change into systems	Quarterly reviews; update toolkits; integrate oversight role to existing operational committees

*for cases with (i) uncertain clinical trajectory or prognosis; (ii) ethical complexity; or (iii) unclear alignment between family and team perspectives.

Abbreviations: AIMD, Aim Ingredients Mechanism and Delivery; NICHD, National Institute of Child Health and Human Disease; BPD, bronchopulmonary dysplasia; Neo, neonatologist; RT, respiratory therapist.

Table S4. LCPR Structured Template, clarifying standard work for various providers

Steps	Person responsible	Task	Goal
1	RT facilitator	o Read out purpose of the LCPR<br>o Estimated BPD risk for the infant<br>o Distribute trend reports (6-8 copies)<br>o Members to introduce to family as necessary	Preparation and setting the stage
2	RT/Neo facilitator	o Share identified issues/ concerns for today's discussion	Keep rounds efficient & focused on weekly goal plans
3	MRP/Delegate	o Summary of hospital course in a nutshell<br>o Active issues<br>o Any additional Issues/ concerns for today's discussion	Brief overview for all participants
4	Bedside RT	o Ventilator status update	Situational awareness
5	RN	o Medications, infusions, IV lines, feeding & Developmental supportive care update	Situational awareness
6	Dietician	o Growth, nutrition concerns	Situational awareness
7	Hemodynamic team member	o PDA, PHT, Cardiac function update	Situational awareness
8	RT Facilitator	o Ask whether anyone has a quick clarifying question or addition	Create shared mental model
9	MRP/Delegate	o Expected course over next 7 days with rationale<br>o Goals for the next week	Empower MRP/Delegate to share their projections based on their observations
10	RT/ Neo facilitator	o Facilitate DISCUSSION	Promote shared understanding, problem solving, and care planning
11	Neo facilitator	o Summarize weekly goals for next 7 days, and seek MRP/everyone's input	Keep rounds efficient & focused on weekly goal plans
12	Neo facilitator	o Anticipated problems & contingency plans<br>o Thresholds for escalation of care & vigilance<br>o Threshold for considering intubation/extubation/other<br>o Readiness criteria for de-escalation (weaning)	Promote emergency preparedness with back up plans
13	RT Facilitator	o We will leave suggested goals in EHR & request MRP/Delegate to update parents on weekly goals	Promote best documentation practice and family engagement
14	RT Facilitator	o EVALUATION- using QR code	Track progress of LCPR & its impact

Aiming for up to 60min. per infant, focus on 2-3 issues / concerns, and facilitated by RT and Neo. Steps 1-8 (5 mins); 9-10 (15 mins); 11-14 (5-10 mins).

Abbreviations: LCPR, longitudinal care planning rounds; RT, respiratory therapist; BPD, bronchopulmonary dysplasia; Neo, neonatologist; RN, registered nurse; PDA, patent ductus arteriosus; PHT, pulmonary hypertension; MRP, most responsible physician; EHR, electronic health record

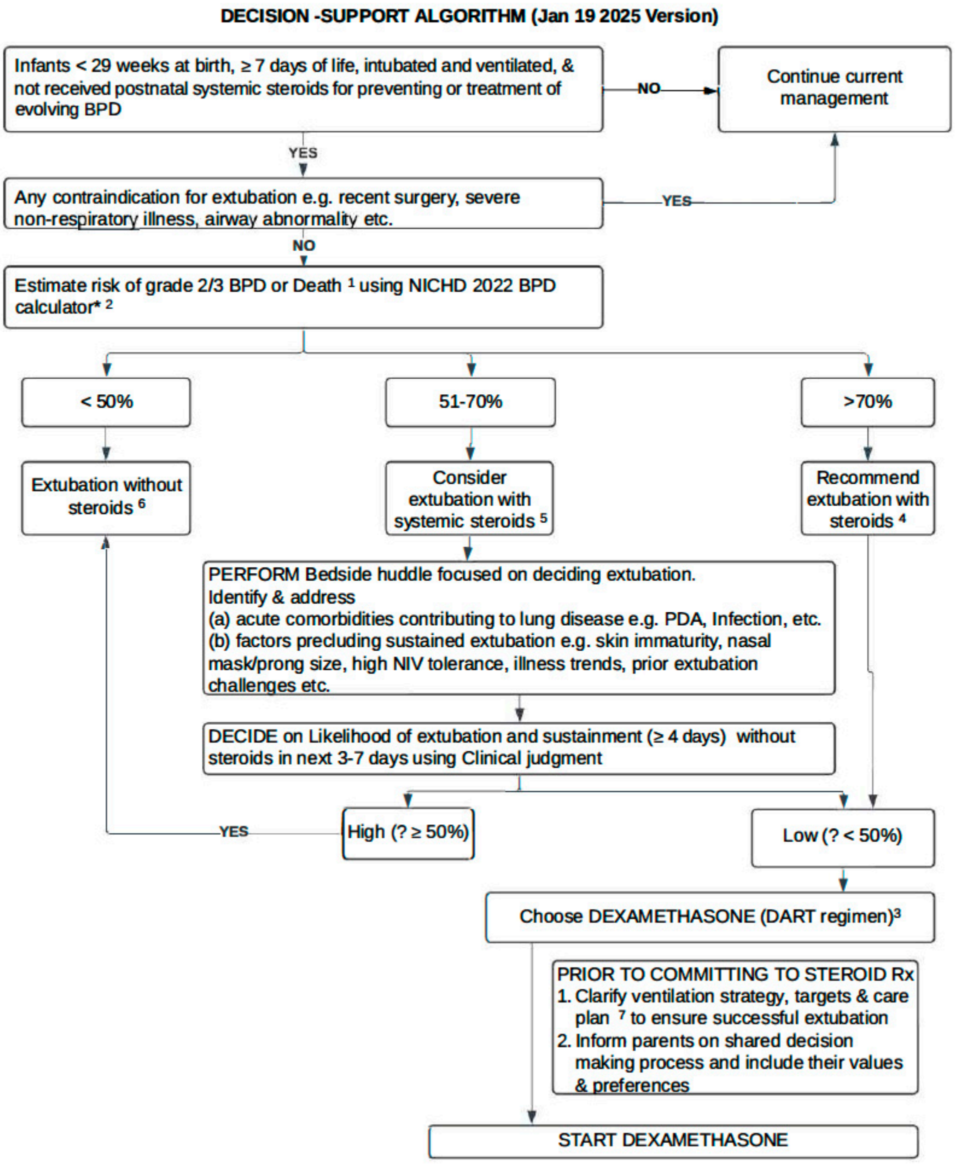

Figure S1. Decision Algorithm for Initiating First Course of sPNS. Abbreviations: sPNS: systemic postnatal steroidsl BPD, bronchopulmonary dysplasia; NICHD, National Institute

File S1. Evidence Brief and Recommendations on Inhaled Steroids

Purpose

To reduce unwarranted variation in the use of systemic and inhaled steroids in extremely preterm infants. This guidance supports appropriate titration of steroids in situations where reducing lung inflammation may proactively prevent intubation.

Evidence: QI studies and current practices from LCPR form the basis of recommendations.

Guidance Statement

Use of systemic or inhaled postnatal steroids to prevent reintubation in extremely preterm infants on non-invasive ventilation after day 7 of life

1. **When to consider steroids**
   Initiate systemic or inhaled postnatal steroids in infants on non-invasive ventilation who meet all of the following criteria:
   - (a) Gradually increasing ventilator requirements.
   - (b) Ventilation trends suggesting progression toward reintubation.
   - (c) No acute reversible cause for increased support (e.g., PDA, infection, abdominal distension, mask leak). The most likely cause is inflammation or rebound inflammation following a prior steroid course.
   - (d) Adequate time (6–24 hours) is available for steroids to take effect.
2. **Treatment options**
   - **Systemic steroids:**
     - Titrated DART regimen (consider extending duration or adjusting dose).
     - STOP-BPD regimen (hydrocortisone).
   - **Inhaled steroids:**
     - Budesonide nebulization.

Note

Use of inhaled steroids in extremely preterm infants on non-invasive ventilation requiring higher levels of support ($FiO_2 \geq 30\%$) for other purposes shown below, requires further discussion.

Goal/Purpose

- Reduce lung inflammation to stabilize histograms and decrease desaturation frequency.
- Facilitate weaning and reduce complications from high NIV pressures (e.g., abdominal distension).
- Potentially lower the risk of reintubation, shorten duration of ventilation, oxygen therapy, and hospitalization.
- Reduce the incidence and severity of BPD.

Abbreviations: QI, quality improvement; LCPR, longitudinal care planning rounds; PDA, patent ductus arteriosus; BPD, bronchopulmonary dysplasia; NIV, non-invasive ventilation

File S2. Evidence Brief and Recommendations on Transpyloric Feeding on EPTs on Ventilatory Support

Purpose

Support consistent, risk-aware selection of infants for transpyloric feeding, and ensure the care team and family review benefits, downsides, and alternatives before initiation.

Background and Rationale

Extremely preterm infants (EPT) who require invasive or non-invasive ventilatory support may develop gastrointestinal problems such as gastric distension, severe reflux, suspected aspiration, or dysmotility. In some infants, these GI issues appear to worsen respiratory status or prevent safe progression of enteral feeding. Transpyloric feeding is used as a strategy to bypass the stomach in carefully selected cases.

Summary of Evidence

The current evidence base is largely observational (case series and retrospective cohorts). Across multiple reports, transpyloric feeding in ventilated EPTs with significant GI-related complications has been associated with:

Potential benefits (reported associations):

- Reduced gastric distension and reflux-related symptoms
- Improved feeding tolerance and ability to advance enteral feeds
- Improved respiratory stability when symptoms are temporally linked to distension or reflux
- In some cases, support for ongoing non-invasive ventilation and avoidance of reintubation

Evidence limitations:

- Predominantly non-randomized designs with selection bias
- Variable definitions, indications, and outcomes across studies
- Complication rates may be under-detected in small series
- Uncertain effect on long-term outcomes

Recommendation

Consider transpyloric feeding only in carefully selected EPTs when GI-related issues are plausibly contributing to respiratory instability or preventing safe feeding progression, and only after a structured risk-benefit discussion with the care team and family.

This is not a routine intervention. It should be treated as a targeted strategy for specific clinical scenarios rather than a default feeding pathway.

Decision Process and Safeguards

Transpyloric feeding should be initiated only after:

- Discussion with **at least two attending neonatologists**, and
- Input from relevant team members (bedside RN, RT, dietitian, and others as appropriate), and
- A documented conversation with the family that includes:
    - Why transpyloric feeding is being considered
    - Expected benefits and realistic uncertainties
    - Risks and potential complications
    - Alternatives, including continued gastric feeding with mitigation strategies

Eligibility Criteria

All the following:

1. **EPT infant**
2. **< 36 weeks postmenstrual age (PMA)**
3. **Receiving ventilatory support**, invasive or non-invasive (including CPAP, NIPPV, NIV-NAVA)
4. Presence of one or more of the following clinical indications:
    - **Respiratory worsening temporally associated with gastric distension**, including increased work of breathing, rising oxygen requirement, or CO_2 retention
    - **Inability to advance feeds** over a clinically meaningful time period due to distension, emesis, or persistent residual concerns
    - **Recurrent need for OG venting/decompression** with ongoing distension despite standard measures

    - **High risk of post-extubation failure** with anticipated extubation to high-level non-invasive support where distension is expected to compromise respiratory mechanics
    - **Suspected aspiration related to severe GERD** contributing to respiratory instability

Required "Pros and Cons" Discussion Points (Minimum)

Before placing a transpyloric tube, explicitly review:

Pros (why it might help):

- Bypasses gastric distension and reflux burden
- May stabilize respiratory course if distension and reflux are driving deterioration
- May improve feeding tolerance and reduce interruptions

Cons (why it might hurt):

- Procedural and device-related complications (malposition, obstruction, mucosal injury, bleeding)
- Potential for serious GI complications (including NEC, perforation, intussusception)
- Tube dislodgement and need for repeat placement
- Uncertain long-term benefit given the observational evidence base
- May distract from addressing root causes (vent settings, fluid strategy, bowel regimen, feeding approach)

Abbreviations: EPT, extreme preterm; GI, gastrointestinal; RN, registered nurse; RT, respiratory therapist; PMA, postmenstrual age; CPAP, continuous positive airway pressure; NIPPV, non-invasive positive-pressure ventilation; NIV-NAVA, non-invasive ventilation with neurally-adjusted ventilatory assist; OG, orogastric; GERD, gastro-esophageal reflux disease; NEC, necrotizing enterocolitis

File S3. Facitator Feedback Form

Please complete this form by hand or electronically and submit to project team co-leads

1. Date: ________________(dd/mm/yyyy)
2. Facilitator name: ______________________
3. Patient in bed spot: T__ - _____
4. # of attendees, including facilitators. Total _______ # of Neos ______

5. Parent(s) present? Yes, No.
6. **Concerns/ issues identified prior to / during rounds:**
   I. ____
   II. ____
   III. ____
   IV. ____
   V. ____
7. **List of identified acute precipitating factors affecting infants trajectory of BPD**

Issue	Management options considered	Preferred option	Rationale
e.g. PDA	*NSAID vs. No Rx*	*No Rx*	*Hemodynamically insignificant*

8. What is the keystone goal for next week?

____

(Stated simply, this is the one goal that, simply by nature of accomplishing it, will support the rest of your goals getting accomplished, including averting or reducing the severity of BPD at 36 weeks. E.g. (i) facilitate extubation, (ii) Prepare for extubation by getting infection/Pulmonary hemorrhage etc. under control, (iii) Maintain extubation or other

Other comments:

____

____

____

Thank you

Abbreviations: Neos, neonatologists; BPD, bronchopulmonary dysplasia; PDA, patent ductus arteriosus; NSAID, non-steroidal anti-inflammatory drug

File S4. Example of documented LCPR summary plan in electronic health record

Baby has been identified with a high risk of developing moderate or severe BPD at 36 weeks gestational age. Ventilation requirements are stabilizing, though significant concerns remain.

1) Overall Comfort Patient appears more comfortable today on fentanyl 4 mcg/kg/hr, with reduced agitation, movement, and bradycardias.

2) Ventilation Trends Significant weaning over the past 2–3 days: PIP 47→34 cmH_2O; MAP 15→12.5 cmH_2O; Ti 0.026→0.020 sec; sigh breaths discontinued; FiO_2 stable at 25–35%. Blood gases remain stable. Positioned lateral due to abdominal distention; supine avoided. Steroids not felt to be beneficial at this time. Lung ultrasound on current HFJV settings showed B-lines throughout with no significant atelectasis.

3) Hemodynamic Status and PDA PDA remains hemodynamically significant with L-R shunt; previously treated with acetaminophen. Diuretics ongoing to minimize pulmonary overcirculation. BP soft but above the 5th percentile. Urine output adequate; no vasoactive support required.

4) Nutrition Receiving TPN; NPO for gut rest given ongoing abdominal concerns.

Management Plan

- Await head ultrasound results. Continue fentanyl for comfort.
- Continue diuretics over the weekend; reassess Monday with repeat echo. Consider transitioning to PRN dosing Monday.
- Wean ventilation as tolerated; target switch to conventional ventilation Sunday to facilitate comfort and skin-to-skin care. Maintain lung-protective strategies until after laparotomy.
- Remain NPO; continue TPN. Coordinate with surgery regarding timing of elective laparotomy and prerequisites. Continue antibiotics; reassess Monday at AMS rounds with ID.

Contingency Planning

- If abdominal deterioration leads to respiratory decline, convene family meeting for advanced care planning and goals of care discussion.
- Prior to any laparotomy (elective or emergent), meet with family to clarify wishes regarding intraoperative findings and intervention.
